# A Mixed-Methods Approach-Based Study of Anaphylaxis Awareness and Educational Needs among Nursing Students

**DOI:** 10.3390/ijerph18179280

**Published:** 2021-09-02

**Authors:** Yoongoo Noh, Insook Lee

**Affiliations:** Department of Nursing, Changwon National University, Changwon 51140, Korea; dobest75@changwon.ac.kr

**Keywords:** anaphylaxis awareness, educational needs, nursing students, mixed-methods

## Abstract

In this mixed-methods study, we identified anaphylaxis awareness among nursing students through a survey, and the needs for anaphylaxis education through focus group interviews (FGIs). Anaphylaxis awareness was surveyed from 10 June–30 July 2018 using a structured questionnaire with 221 junior or senior nursing students. To assess the needs for anaphylaxis education, 14 senior nursing students were interviewed using FGIs from 10–22 June 2018. Quantitative data were analyzed descriptively using SPSS, and qualitative data were assessed using thematic analysis. Nursing students’ awareness of anaphylaxis was identified by correct answers to items concerning symptoms (33.0%), causes (64.6%), and treatments (55.8%), with an overall average of 51.5%. The thematic analysis identified four themes reflecting students’ educational needs: the need for anaphylaxis education, various education methods, field training-based study of educational content, and repeated education. Nursing students’ anaphylaxis awareness is low. Their specific needs derived in this study for anaphylaxis education should draw special attention when planning nursing curriculum. Incorporating these findings in future education programs may promote students’ confidence in treating anaphylaxis.

## 1. Introduction

Anaphylaxis is a systemic allergic reaction that occurs for various reasons, and it can lead to death owing to rapidly developing diverse symptoms in several organs over a short period [1,2]. When symptoms appear, proper and quick emergency treatment is crucial, such as accurate diagnosis and injection of epinephrine [1]. The recent increase in the number of patients experiencing anaphylaxis worldwide has resulted in a six-fold increase in the incidence of anaphylaxis since 1992, with a recurrence rate of one in three [3,4].

Anaphylaxis awareness refers to the degree of understanding of the causes, symptoms and treatment of anaphylaxis. In a survey of 15 university hospitals from 2007 to 2011, 42% of patients experiencing anaphylaxis were classified as serious, raising the importance of anaphylaxis treatment [5]. Several studies have identified the ability to recognize anaphylaxis and use epinephrine in nurses, emergency rescuers, primary care doctors, medical workers, and school nurses, professionals who are among the most likely to encounter patients experiencing anaphylaxis [1,6,7,8,9]. In those studies, there were significant differences in perception among doctors, nurses, and healthcare personnel regarding anaphylaxis, epinephrine dose, and administration methods [1,6,8,9]. Only a quarter of the medical workers had heard about epinephrine injections [6], and this knowledge was less common in nurses than in doctors [1,6,8]. Although anaphylaxis education is routinely included in the nursing curriculum as part of pathology, adult health nursing, or emergency care in most nursing colleges, nursing students’ knowledge of anaphylaxis is very low and should be supplemented by education [7].

Until now, efforts to increase the awareness of anaphylaxis and its treatment were mostly directed at doctors, nurses, medical workers, health teachers, and medical students. Thus, there is a dearth of research on nursing students [7]. It has also been noted that anaphylaxis education for nursing students is insufficient [10].

Therefore, it is necessary to identify nursing students’ anaphylaxis awareness from the perspective of nursing education. This, along with the identification of the educational need regarding anaphylaxis, will provide the basis for improving the nursing curriculum. This study aims to identify awareness about the causes, symptoms, and treatments of anaphylaxis, as well as its educational needs of nursing students using mixed-methods research. The purpose of the research is as follows:

(1) Identify the anaphylaxis awareness of nursing students.
(2) Confirm the educational needs for anaphylaxis through the nursing students’ FGI.

## 2. Materials and Methods

### 2.1. Study Design

This study employed a mixed-methods design using both quantitative researches to identify the students’ awareness of anaphylaxis and qualitative research to identify their educational needs through focus group interviews (FGIs). Mixed-methods research has the advantage of overcoming and complementing the shortcomings of quantitative research. Because of the diversity of data collection, it has high feasibility and helps researchers better understand participants [11]. Since qualitative research has the advantage of discovering phenomena that are hidden from quantitative research, the exploration of students’ educational needs through thematic analysis along with anaphylaxis awareness will elucidate the educational status of anaphylaxis.

### 2.2. Participants

This study was conducted with nursing students from three nursing colleges that were deemed eligible for anaphylaxis training in Changwon and Daegu Cities, South Korea. The criteria for selecting participants were: [1] they understood the purpose of the study and the research procedure and voluntarily provided written consent; and [2] they were nursing students in their third or fourth year of college who had taken one or more practice courses. As calculated in G*Power 3.1 (Franz Faul, Düsseldorf, Germany), the minimum preferred sample size was estimated to be 210 using a 90% confidence level and 90% power. We recruited 221 students for our quantitative study, including 14 students who participated in the qualitative study.

For qualitative study, a focus group was recruited from among fourth-year students in the nursing department of the institution to which the researcher belongs, considering that the group should consist of participants with common characteristics in relation to the theme. Participants were selected from those who were able to give clear and candid opinions on anaphylaxis education after a public notice, and who understood the purpose of the study and agreed to participate. For convenience, we recruited 14 students: 12 women and two men. Considering that discussion is most active in a focus group with no more than six to 10 members [12], we split the participants into two groups of the same size, each having seven members. Participants were thus free to express their opinions in a comfortable atmosphere.

### 2.3. Measures

This study used structured survey tools for the quantitative component and semi-structured interview questions for the qualitative component.

#### 2.3.1. Anaphylactic Awareness Measurement

Measurements were performed using anaphylaxis questions provided by the AAAAI (American Academy of Allergy, Asthma and Immunology) [13]. The tool is accessible online, was translated into Korean, and was used with permission from the Academy. It consists of 10 questions: 3 on causes, 2 on symptoms, and 5 on treatments. One point was given if correct and 0 points if incorrect, with the total score ranging from 0 to 10. Higher scores indicated higher awareness of anaphylaxis. Two researchers conducted the primary translation, and a third researcher performed a back translation to check for accuracy. For the translated questions, two nursing professors were consulted, and the calculated index of content validity was 0.90.

#### 2.3.2. FGI Questions on the Educational Needs Regarding Anaphylaxis

Semi-structured questions were constructed by two researchers considering the study aims. The main question was, “What do you think about anaphylaxis education?” In more detail, the question framework was whether anaphylaxis education is necessary and what is required for it. Although a semi-structured interview was used, sufficient time was provided to freely discuss questions or other opinions that were deemed necessary during the interview process.

### 2.4. Data Collection 

Data were collected by two researchers who explained the purpose and aim of the study to the heads of the nursing departments of the three nursing colleges and requested cooperation. Overall, 230 students were surveyed, and data were collected from 10 June-30 July 2018 using self-reporting questionnaires. The final analysis was conducted on 221 respondents, excluding seven who had omitted answers and two who had provided insincere responses (effective recovery rate = 96.1%).

After completion of the quantitative study, 14 students who agreed to participate in the qualitative study completed the FGIs. They were notified about the interview time prior to the meeting. The interviews were conducted in a quiet and isolated seminar room to make students feel comfortable and relaxed. The interviews were recorded and then transcribed verbatim. Interviews lasted approximately 1–1.5 h per group. The interviews were conducted based on the question categories presented by Kruger and Casey [14]: start/introduction/transition/core/finish process (Table 1). Recordings and on-site notes were taken throughout the interviews, and participants’ attitudes and characteristics were described. In the final stage of the interview, the interviewers revisited and clarified any unclear comments.

Data were collected from 10–22 June 2018, and these data were organized from 23 June–30 July 2018 by transcribing the recordings and comparing them with the on-site notes. Until 30 August 2018, researchers continued to read and analyze the original data repeatedly. If there were any differences in interpretation among researchers, the process of reading and reviewing the original data was repeated until consensus was reached.

### 2.5. Analysis

Quantitative data were analyzed using SPSS/WIN 25.0 (SPSS; IBM, Armonk, NY, USA), and qualitative data from FGIs were thematically analyzed as follows:

(1) Means, standard deviations, numbers, and percentages were calculated for participants’ general characteristics and awareness of anaphylaxis.
(2) Qualitative analysis of interview content was conducted using the thematic analysis of Braun and Clarke [15]. First, two researchers carefully read all data collected through interview records and on-site memos to fully understand the data; then, they selected meaningful data and underlined them to indicate meanings or patterns. Second, the underlined data were reviewed, and the characteristics of the data were coded. Third, the common codes were collected and classified according to potential themes by the same researchers. Themes and subthemes were derived through this step. Fourth, the identified themes were modified to reflect the overall data and compared with subthemes. Fifth, the derived themes and subthemes were clearly named. Lastly, the researchers performed the final analysis and data interpretation.

### 2.6. Ensuring the Data Quality to Generate Authenticity of the Qualitative Study

In order to be qualified as a qualitative researcher, two researchers attended more than 10 seminars on qualitative research methodology. With the experience of publishing three and one qualitative research within the last five years, respectively, it was thought that the research was suitable for them to carry out. 

We sought to ensure the quality of qualitative research in accordance with credibility, transferability, dependability, and confirmability, which are the standards of scientific research argued by Lincoln and Guba [16]. 

Credibility refers to the degree to which the researcher has confidence in the results. We tried to interpret the participants’ statements at face value to increase credibility. The interview transcripts and analysis results were shown to the participants to ensure that they were consistent with their statements. Transferability means the degree to which generalization is possible. To secure transferability, researchers described the data in depth (‘thick description’ in other words) so that the findings derived from the interviews could be applied to other nursing students. Dependability is whether the study was conducted in a consistent and reproducible manner. The researchers sought to ensure consistency in the study by generating themes from the data according to the method of thematic analysis and making corrections to theme categorization. Confirmability refers to the degree to which data are processed and interpreted in a non-biased and neutral manner. In order to maintain confirmability, we continuously recorded and compared the interview data and topics with each other throughout the study period to ensure that the researchers did not have any preconceptions, assumptions, or prejudices about the study.

## 3. Results

### 3.1. Quantitative Results

Participants’ demographic data and awareness level are shown in Table 2. Most had received lecture-based education; however, no one had hands-on training. Nearly all agreed that education on anaphylaxis was necessary. There was a difference in the anaphylaxis awareness score by college: the mean score of college A was significantly higher than that of colleges B and C.

Regarding students’ awareness of anaphylaxis, the overall average concerning the correct awareness of anaphylaxis was 51.1% (Table 3; subcategories ranged from 33–64.6%). Regarding symptoms, half the students chose the correct answer to Q1 (Can symptoms of anaphylaxis occur either shortly after or hours after allergen exposure?). However, for Q2 (Can the anaphylaxis reaction be as simple as developing a rash?), the percentage of correct answers dropped sharply (15.8%). When asked if anaphylaxis can occur from normal edible foods (Q3), 95.0% answered correctly. When asked about the least likely cause of anaphylaxis (Q4; see Table 3), very few students (5.0%) answered correctly. Concerning treatments, most students responded correctly to Q9 (Does anaphylaxis always require medical treatments? 86.9%) and Q6 (regarding the optimal time of epinephrine administration; 85.5%). When asked about the epinephrine injection site (Q7), 46.6% chose correctly (i.e., thigh). For Q8 (Are antihistamines and steroids good substitutes for epinephrine?), only 24.0% answered correctly.

### 3.2. Qualitative Results

Nursing students’ educational needs regarding anaphylaxis generated four main themes: “Necessity of anaphylaxis education”, “Necessity of various education methods”, “Necessity of field training-based study of educational content” and “Necessity for repeated education”.

#### 3.2.1. Necessity of Anaphylaxis Education

Students said that, despite the rarity of its occurrence, anaphylaxis can cause serious problems for patients and that the current education does not keep up with the growing increase in prevalence. They consistently stressed the importance of educational programs as follows:

*“…I know that children with anaphylaxis are on the rise these days…If you find someone who is experiencing anaphylactic shock, you should inject epinephrine, but you do not know how much and where to give it…I do not think I can apply it in real life even if I have some knowledge…I think we need to learn more.”*

(P3)

*“It was wintertime in my second year of elementary school, when I had sudden difficulty breathing…As a nursing student, I hope I could acquire knowledge and skills to cope with such an emergency situation. Education is of utmost importance.”*

(P5)

#### 3.2.2. Necessity of Various Education Methods

Students said that supplementing lectures with photos and videos would make the lectures even richer. They noted the value of case presentations, role playing, simulations, and clinical practice adopting hands-on manipulation of emergency kits. They also asked for project-based learning (PBL) courses where they could share and discuss each other’s experiences and receive feedback from professors.

*“It would be memorable if [an] education program is implemented in this sequence: systematic acquisition of basic concepts, followed by more lively understanding of the contents with the aid of video or other multimedia materials, and finally rehearsal in simulated situations.”*

(P7)

*“…Topic discussions or PBL classes were the most impressive, leaving the longest lasting memory.”*

(P5)

#### 3.2.3. Necessity of Field Training-Based Study of Educational Content 

Students expressed great concern regarding the education aimed at the appropriate management and prevention of anaphylaxis. It should be practical and comprehensive enough to cover the whole spectrum of anaphylaxis management: causes, symptoms, choice of drugs and their dosage, administration pathways, use of emergency kits, customized nursing to fit the clinical condition, and post-medication monitoring and nursing.

*“…symptoms, patients’ physical changes, quick action, treatments, dosage, etc. I want to learn how to deal clinically. Case studies will be helpful.”*

(P8)

*“Let’s suppose that an anaphylaxis patient fell right in front of us…What [do I] do first? How [do I] do it step-by-step? I want to learn the causes, symptoms, how to deal with them, [and] how to manage them in the future.”*

(P13)

#### 3.2.4. Necessity of Repeated Education

Students wanted at least one regular training session a year because they could forget it over time.

*“We learned a little in many subjects by the 2nd year in college; but now we are in the 4th year, and I have only faint memories…I wish we had the chance of having periodic and serial education programs adjusted to each school year so we can learn more in depth…”*

(P1)

## 4. Discussion

In this study, nursing students’ anaphylaxis awareness and anaphylaxis education needs were confirmed using mixed-methods research.

Students’ overall awareness of anaphylaxis (51.1%; 64.6% for causes, 55.8% for treatments, and 33.0% for symptoms) fell between that seen in nurses (56.3%) and that seen in medical students (47.7%) in a prior study (1). Concerning symptoms, although direct comparison is limited owing to the scarcity of previous studies, the rate in this study was lower than what has been seen among nurses (65.8%) and medical students (45.6%) (1). This gap is even greater when it comes to Q2 (Can the anaphylaxis reaction be as simple as developing a rash?): 15.8% correct rate in this study versus 59.2% (nurses) and 43.8% (medical students) in a prior study [1]. These results should be specifically addressed in education as an indication that nursing students do not fully understand anaphylaxis symptoms.

Awareness of causes showed 64.6% accuracy, higher than the 50.9% for medical students and 61.3% for nurses in a prior study [1]. While nearly all participants knew whether anaphylaxis could occur from foods that are commonly eaten, only 5.0% were aware of the least likely cause of anaphylaxis. This was roughly similar to the 98.2% and 1.2% for nurses and 76.1% and 5.7% for medical students, respectively, seen previously [1]. The correct answer rate was 93.7% for the question, “If the allergic reaction was light in the past, there would be no life-threatening allergic reaction in the future.” This was higher than the 84.7% and 70.9% for nurses and medical students, respectively, in a prior study (1). The least likely cause of anaphylaxis, as well as the most common cause, should also receive special attention when educators plan the curriculum.

Concerning awareness of treatments, nursing students had a correct answer rate of 55.8%, higher than the 49.5% for nurses and 46.7% for medical students in a prior study [1]. In particular, the questions with a high correct answer rate were “anaphylaxis should always be treated with medication” (86.9%) and “epinephrine administration should be administered in the early stages of the symptom” (85.5%). Questions with low correct answer rates were “antihistamines and steroids are good substitutes for epinephrine” (24.0%) and “the best way to manage one’s condition in the event of anaphylaxis risk” (36.2%).

In summary, the overall anaphylaxis awareness of nursing students was lower than that of nurses but higher than that of medical students, and it differed per causes, symptoms, and treatments. Nursing students thus need to be educated about the causes, symptoms, and treatments that they were typically unaware of. In contrast, for certain items, nursing students showed higher correct answer rates than did nurses and medical students. We think this is the effect of repetitive education, as shown by a previous study reporting that three-month post-education scores were higher than six-month post-education scores [6].

Regarding educational experiences, 89.2% of the students received lectures on anaphylaxis; however, none were trained in practice. The high rate of lecture but an anaphylaxis awareness of just around 50% indicates that the lectures alone do not necessarily translate into increased awareness of anaphylaxis. Through clinical practice, which seems to be essential, students would have the opportunity to integrate theory and practical knowledge [17].

In our qualitative component, the thematic analysis yielded four themes. The first theme highlighted the necessity for anaphylaxis education. That is, despite the growing occurrence of anaphylaxis and the urgent need for quick response in emergency situations, there has not been enough education. Thus, students felt more education is needed.

Recently, the prevalence of anaphylaxis has increased worldwide, raising alertness and stimulating research on its treatment and care guidelines [18]. Nurses play a central role in assessing and reporting changes in patients and administering medications. Appropriate follow-up management and advice should also be given to reduce the risk of recurrence [19]. Nursing students, however, scored low on anaphylaxis knowledge and primary choice drugs [7], and only 35.2% of school nurses were found to have been educated about food anaphylaxis [20]. In this context, anaphylaxis should be included in the curriculum to promote healthcare providers in responding quickly to changing medical environments and to improve patient safety and nursing quality.

The second theme comprised the necessity of various education methods. Students listed audiovisual education, kits and dummies-based practice, case analysis and discussion, and simple role playing in addition to basic concepts as effective educational methods because they are long-remembered and efficient.

The benefits of applying various educational methods have been studied previously. Meechan et al. [21] reported improved knowledge of pharmacology in nursing students by introducing an integrated approach to pharmacology. Through simulation training, students in anesthesia care who initially had fear of anaphylaxis development, displayed improvements in critical thinking, decision making, confidence, and clinical preparedness [22]. Since such diverse educational approaches have positive educational effects, it is necessary to introduce various educational methods to improve the knowledge and practical skills of nursing students. Conceptual learning, audiovisual training, kits and dummies-based practice, simulation, hands-on experience sharing, and case analysis and discussion based on each academic stage may improve students’ anaphylaxis awareness and practical ability.

The third theme concerned the necessity of field training-based study of educational content. In addition to comprehensive education covering post-treatment care and long-term management for prevention, students showed special interest in knowing the specific treatment processes such as treatment prioritization, using emergency kits, drug administration, and nursing tailored to individual patients. Anaphylaxis is a medical emergency that requires prompt assessment and intervention to reverse the cascade of reactions leading to death; therefore, most academic societies recommend treating anaphylaxis under the guidance of a standardized protocol [23]. Nonetheless, the management of anaphylaxis is not always straightforward. As it happens in other clinical areas, anaphylaxis manifestation may vary over a wide range of spectra and may not present with stereotyped patterns, mandating customized implementation of the protocol [24]. The best way to understand this complex decision process in anaphylaxis treatment would be systematic, specific, and practical education. 

The final theme concerned the necessity for repeated education. A previous study showed that more than 85% of physicians in a tertiary hospital emergency department answered correctly when asked about treatments and routes of drug administration for anaphylaxis [8]. In contrast, about 57% of interns, medical students, and nursing students correctly chose epinephrine as the first-line drug for treating anaphylaxis [7]. This difference could be attributed to the relatively common exposure to anaphylaxis in the emergency department. Thus, it is understandable that students, having little chance of confronting anaphylaxis situations, want regular education. Our students stated that education once a year would be preferable to reinforce their memory. There has not been much study on the optimal period of education. Topal et al. [25] found that interns’ epinephrine auto-injection skills were sustained for three months after basic training and noticeably decreased when assessed after six months. This may provide some clues as to the optimal period of anaphylaxis education (e.g., every six months).

Finally, we admit that there are some limitations in this study. The curriculum contents may vary among nursing colleges; thus, there is a limit to generalization in conducting research with nursing college students in confined regions. In a similar vein, participants were recruited only from one college for the interview about their educational needs. Further, first- and second-year students’ anaphylaxis awareness could not be evaluated because they were not included in this study. Subsequent studies encompassing a wider group of participants with various regional and educational backgrounds may help address these concerns. 

We also understand that some readers might have had concerns regarding the need for a pilot study before using the questionnaire tools. The questionnaire for quantitative analysis in this study did not aim to collect opinions or graded responses (e.g., pain score) from respondents. It simply comprised questions with T or F values. It is very unlikely that college students could have misunderstood these simple questions provided by AAAAI. Thus, we did not think that a pilot study was warranted. 

The strength of this study is that we systematically identified anaphylaxis awareness among nursing students, and confirmed their level of awareness for three clinical categories: cause, symptoms, and treatment. In addition, through FGIs, four themes were derived by confirming students’ varied needs for anaphylaxis education.

## 5. Conclusions

We studied anaphylaxis awareness and anaphylaxis educational needs among nursing students. Students’ awareness was lower than that of nurses but higher than that of medical students, and it was lower for symptoms than for causes and treatments. 

Regarding students’ need for anaphylaxis education, nearly all agreed that it was necessary. Their specific requests yielded four themes derived from our thematic analysis: necessity for anaphylaxis education, necessity of various education methods, necessity of field training-based study of educational content, and necessity for repeated education.

The low level of anaphylaxis awareness, coupled with the ardent requests for practical education, highlight the need to develop a more advanced and well-woven program for the nursing students, especially in this era of COVID-19 pandemic.

## Figures and Tables

**Table 1:** Essential research questions.

Order	Contents
Start	Please introduce yourself briefly.
Introduction	What do you think about anaphylaxis education?
Transform	Do you think anaphylaxis education is necessary?
Key question	What do you think is necessary for anaphylaxis education?
Concluding question	Lastly, please comment on anaphylaxis education.

**Table 2:** Demographic characteristics and anaphylaxis awareness differences among participants *(n* = 221).

Variable	Category	*n* (%)	Awareness Score Mean ± SD	*t*/F (*p*)
Sex	Male	15 (6.8)	5.33 ± 1.18	−0.26 (0.793)
	Female	204 (93.2)	5.42 ± 1.26	
	Total	219 (100.0)	5.42 ± 1.25	
School years	3rd	118 (53.9)	5.51 ± 1.43	1.22 (0.225)
	4th	101 (46.1)	5.31 ± 1.01	
	Total	219 (100.0)	5.42 ± 1.25	
Age (years)	≤20	53 (24.4)	5.62 ± 1.53	0.97 ^†^ (0.381)
	21	88 (40.6)	5.31 ± 1.24	
	≥22	76 (35.0)	5.41 ± 1.06	
	Total	217 (100.0)	5.42 ± 1.26	
Learning experience regarding anaphylaxis	Lecture	182 (89.2)	5.48 ± 1.28	5.38 (0.072)
	Practice	0 (0.0)	-	
	None	22 (10.8)	4.96 ± 1.33	
	Total	204 (100.0)	5.42 ± 1.29	
Necessity of education	None	7 (3.2)	5.29 ± 0.76	−0.28 (0.782)
	Yes	212 (96.8)	5.42 ± 1.27	
	Total	219 (100.0)	5.42 ± 1.25	
College	College A	56 (25.3)	6.11 ± 1.12	14.12 ^†^ (<0.001)
	College B	86 (38.9)	5.06 ± 1.40	
	College C	79 (35.8)	5.24 ± 1.04	
	Total	221 (100.0)	5.39 ± 1.28	

Note: Missing values were excluded. ^†^ Brown–Forsythe test. SD = standard deviation.

**Table 3:** The percentages of correct answers to the questionnaire (*n* = 221).

Questionnaire Item	Correct *n* (%)	Sub-Category (%)
Q1. Symptoms of anaphylaxis can occur: (1) Shortly after coming into contact with an allergen (2) Hours after coming into contact with an allergen (3) Either of the above	111 (50.2)	Symptoms (33.0)
Q2. An anaphylactic reaction can be as simple as developing a rashafter exposure to an allergen. (1) True (2) False	35 (15.8)	
Q3. Anaphylaxis can occur from eating common foods such as milk, eggs, or shellfish. (1) True (2) False	210 (95.0)	Causes (64.6)
Q4. Which of these are not likely to cause anaphylaxis: (1) Medications (2) Pollen (3) Latex (4) Exercise (5) Stinging Insects	11 (5.0)	
Q5. If you had a mild allergic reaction to an allergen in the past, then you are not at risk for a life-threatening reaction in the future. (1) True (2) False	207 (93.7)	
Q6. Epinephrine should be given early in symptoms of anaphylaxis. (1) True (2) False	189 (85.5)	Treatments (55.8)
Q7. Epinephrine should be injected into the: (1) Arm (2) Thigh (3) Buttocks	103 (46.6)	
Q8. Antihistamines and corticosteroids are good substitutes for epinephrine in treating anaphylaxis. (1) True (2) False	53 (24.0)	
Q9. Anaphylaxis always requires medical treatment. (1) True (2) False	192 (86.9)	
Q10. If you are at risk for anaphylaxis, the best way to manage your condition is: (1) Avoid allergens that trigger symptoms (2) Carry auto-injectable epinephrine (3) Know how to use epinephrine (4) Develop an anaphylaxis action plan (5) All of the above	80 (36.2)	
		Total (51.5)

Note: Q1–Q10 are 10 questions taken from the Anaphylaxis Quiz (http://aaaai.org accessed on 13 December 2017).

## Data Availability

The data used and/or analyzed during the current study are available from the corresponding author on request.
